# Supplementary material for: Improved Split TEV GPCR β-arrestin-2 Recruitment Assays via Systematic Analysis of Signal Peptide and β-arrestin Binding Motif Variants
Source: Biosensors (Basel). 2022 Dec 29;13(1):48. doi: 10.3390/bios13010048 (PMC9855867; doi:10.3390/bios13010048)
Supplement: Supplementary file 1 [file biosensors-13-00048-s001.zip › biosensors-2064368-supplementary.pdf]

biosensors

Supplementary Material

Improved Split TEV GPCR β-arrestin-2 Recruitment Assays via Systematic Analysis of Signal Peptide and β-arrestin Binding Motif Variants

Yuxin Wu [1,†], Isabelle V. von Hauff [1,†], Niels Jensen [2], Moritz J. Rossner [2,3], Michael C. Wehr [1,3,*]

[1] Research Group Cell Signalling, Department of Psychiatry and Psychotherapy, University Hospital, Ludwig Maximilian University of Munich, Nussbaumstr. 7, 80336 Munich, Germany
[2] Section of Molecular Neurobiology, Department of Psychiatry and Psychotherapy, University Hospital, Ludwig Maximilian University of Munich, Nussbaumstr. 7, 80336 Munich, Germany
[3] Systasy Bioscience GmbH, Balanstr. 6, 81699 Munich, Germany
* Correspondence: michael.wehr@med.uni-muenchen.de
† These authors contributed equally to this work.

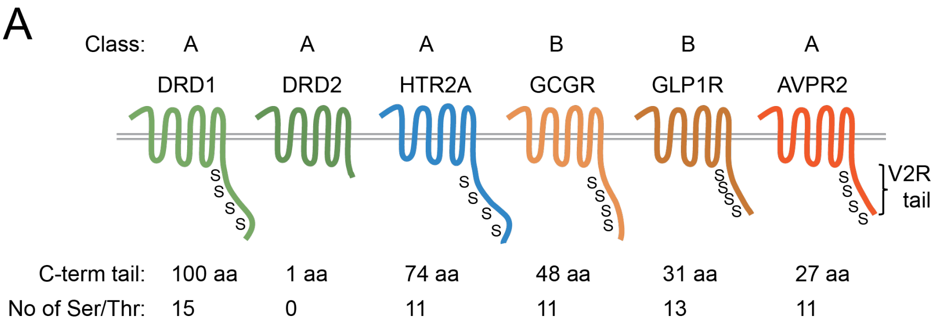

B

DRD1:
CYRLCPATNNAIETVSINNNGAAMFSSHHEPRGSISKECNLVYLIPHAVGSSEDLKKEEAAG
IARPLEKLSPALSVILDYDTDVSLEKIQPITQNGQHPT

DRD2:
C

HTR2A:
QYKENKKPLQLILVNTIPALAYKSSQLQMGQKKNSKQDAKTTDNDCSMVALGKQHSEEASKD
NSDGVNEKVSCV

GCGR:
TSNHRASSSPGHGPPSKELQFGRGGGSQDSSAETPLAGGLPRLAESPF

GLP1R:
SMKPLKCPTSSLSSGATAGSSMYTATCQASCS

AVPR2:
GRTPPSLGPQDESCTTASSSLAKDTSS

Figure S1. Split TEV GPCR constructs were properly expressed in HEK-293 cells. (**A**) GPCRs tested in this study. Note that GPCRs had different C-terminal tails with varying numbers of phosphorylatable serine and threonine residues that when phosphorylated impacted on β-arrestin-2 binding. (**B**) Protein sequences of the C-terminus tails of the GPCRs shown in (**B**). Serine and threonine residues are highlighted in red.

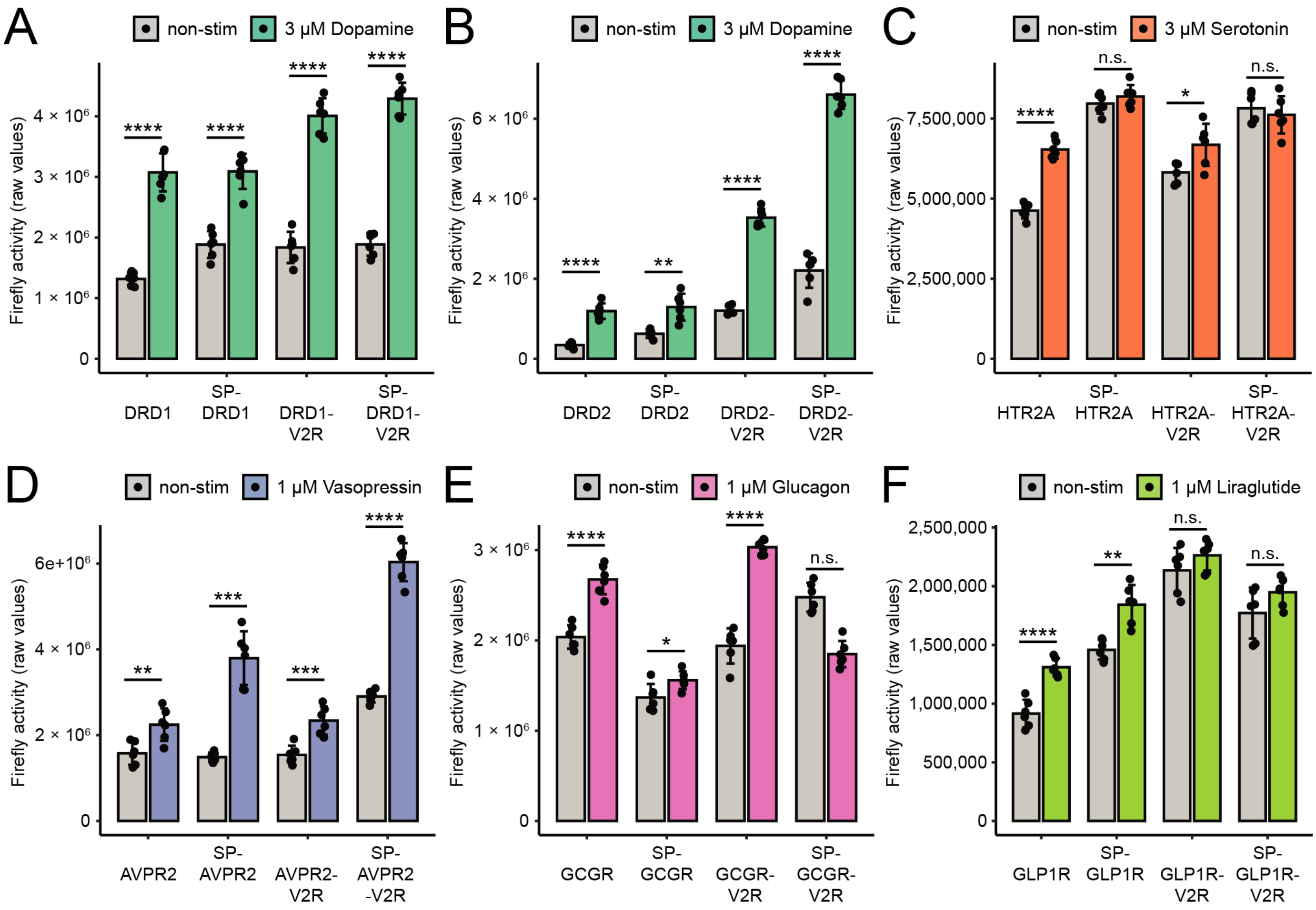

Figure S2. Split TEV GPCR b-arrestin-2 recruitment assays in HeLa cells. (**A-F**) Luciferase end-point assays for DRD1 (**A**), DRD2 (**B**), HTR2A (**C**), AVPR2 (**D**), GCGR (**E**), and GLP1R (**F**). All assays were conducted in a 96-well format. Assays were stimulated for 6 h using cognate agonists. Bar graphs display means, error bars represent s.d. with six replicates per conditions. Two-tailed Student's *t* test was used to determine the *p*-values for treatment versus control. *, $p \leq 0.05$; **, $p \leq 0.01$; ***, $p \leq 0.001$; ****, $p \leq 0.0001$, n. s., not significant.

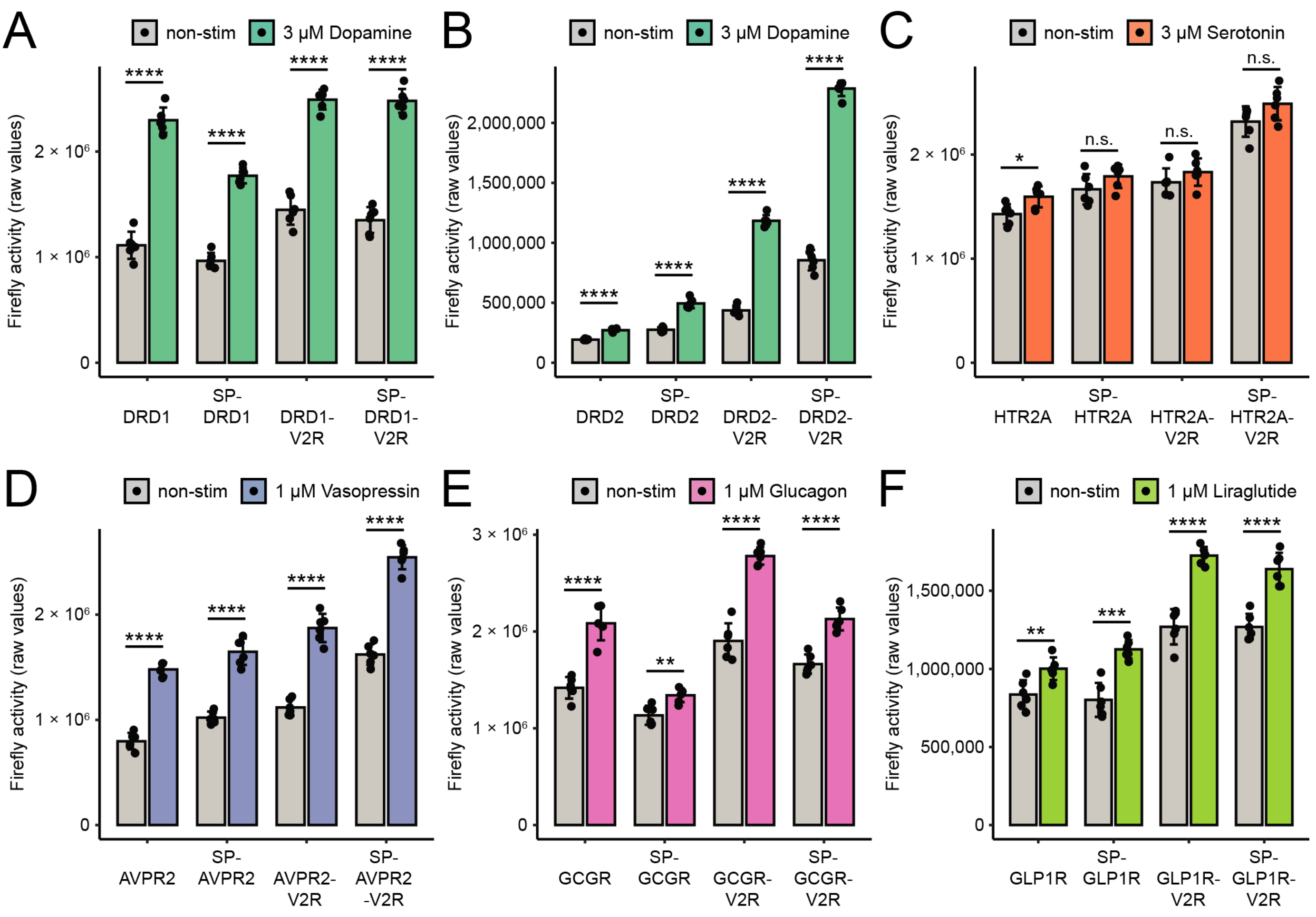

Figure S3. Split TEV GPCR β-arrestin-2 recruitment assays in U-2 OS cells. (**A-F**) Luciferase end-point assays for DRD1 (**A**), DRD2 (**B**), HTR2A (**C**), AVPR2 (**D**), GCGR (**E**), and GLP1R (**F**). All assays were conducted in a 96-well format. Assays were stimulated for 6 h using cognate agonists. Bar graphs display means, error bars represent s.d. with six replicates per conditions. Two-tailed Student's *t* test was used to determine the *p*-values for treatment versus control. *, $p \leq 0.05$; **, $p \leq 0.01$; ***, $p \leq 0.001$; ****, $p \leq 0.0001$, n. s., not significant.

Table S1. Plasmid information (plasmids are available at Addgene).

Addgene ID	Plasmid Name	Target/Insert	RefSeq ID	kDa, calculated (ORF)
194358	pcDNA3_DRD1-NTEV-TCS-GV-2xHA	DRD1	NM_000794	102.5
194359	pcDNA3_SP-DRD1-NTEV-TCS-GV-2xHA	DRD1	NM_000794	105.6
194360	pcDNA3_DRD1-V2R-NTEV-TCS-GV-2xHA	DRD1	NM_000794	105.7
194361	pcDNA3_SP-DRD1-V2R-NTEV-TCS-GV-2xHA	DRD1	NM_000794	108.8
194362	pcDNA3_DRD2-NTEV-TCS-GV-2xHA	DRD2	NM_016574	100.6
194363	pcDNA3_SP-DRD2-NTEV-TCS-GV-2xHA	DRD2	NM_016574	103.7
194364	pcDNA3_DRD2-V2R-NTEV-TCS-GV-2xHA	DRD2	NM_016574	103.8
194365	pcDNA3_SP-DRD2-V2R-NTEV-TCS-GV-2xHA	DRD2	NM_016574	106.8
194366	pcDNA3_HTR2A-NTEV-TCS-GV-2xHA	HTR2A	NM_000621	105.8
194367	pcDNA3_SP-HTR2A-NTEV-TCS-GV-2xHA	HTR2A	NM_000621	108.9
194368	pcDNA3_HTR2A-V2R-NTEV-TCS-GV-2xHA	HTR2A	NM_000621	112
194369	pcDNA3_SP-HTR2A-V2R-NTEV-TCS-GV-2xHA	HTR2A	NM_000621	115.1
194370	pcDNA3_AVPR2-NTEV-TCS-GV-2xHA	AVPR2	NM_000054	93.5
194371	pcDNA3_SP-AVPR2-NTEV-TCS-GV-2xHA	AVPR2	NM_000054	96.6
194372	pcDNA3_AVPR2-V2R-NTEV-TCS-GV-2xHA	AVPR2	NM_000054	96.7
194373	pcDNA3_SP-AVPR2-V2R-NTEV-TCS-GV-2xHA	AVPR2	NM_000054	99.8
194374	pcDNA3_GCGR-NTEV-TCS-GV-2xHA	GCGR	NM_000160	107.2
194375	pcDNA3_SP-GCGR-NTEV-TCS-GV-2xHA	GCGR	NM_000160	110.3
194376	pcDNA3_GCGR-V2R-NTEV-TCS-GV-2xHA	GCGR	NM_000160	110.4
194377	pcDNA3_SP-GCGR-V2R-NTEV-TCS-GV-2xHA	GCGR	NM_000160	113.5
194378	pcDNA3_GLP1R-NTEV-TCS-GV-2xHA	GLP1R	NM_002062	106.5
194379	pcDNA3_SP-GLP1R-NTEV-TCS-GV-2xHA	GLP1R	NM_002062	109.6
194380	pcDNA3_GLP1R-V2R-NTEV-TCS-GV-2xHA	GLP1R	NM_002062	112.7
194381	pcDNA3_SP-GLP1R-V2R-NTEV-TCS-GV-2xHA	GLP1R	NM_002062	115.8
194382	pcDNA3.1_Zeo_ARRB2-1-383-CTEV-2xHA	ARRB2	NM_004313	58.7
194383	pGL4_10xUAS-MLPmin-luc2	10xUAS-MLPmin-luc2	not applicable	not applicable
194384	pGL4_CRE-CMVmin-luc2	6xCRE-CMVmin-luc2	not applicable	not applicable
194385	pcDNA3_attR1-ORF-attR2-NTEV-TCS-GV-2xHA_DEST	Gateway Destination vector	not applicable	not applicable
194386	pcDNA3_attR1-ORF-attR2-V2R-NTEV-TCS-GV-2xHA_DEST	Gateway Destination vector	not applicable	not applicable

Table S2. Oligonucleotides used for cloning.

Oligo name	Sequence	Target	Purpose
FLAG-DRD1_fwd	GGGGACAAGTTT-GTACAAAAAAGCAGGCT	DRD1	Addition of signal peptide, first PCR, forward primer
FLAG-DRD2_fwd	GACTACAAGGACGATGATGAC-GCCAGCATCGATATGGATCCAC-TGAATCTGTCCTG	DRD2	Addition of signal peptide, first PCR, forward primer
FLAG-HTR2A_fwd	GACTACAAGGACGATGATGAC-GCCAGCATCGA-TATGCAATTAAATGATGACAC-CAGGCTCTAC	HTR2A	Addition of signal peptide, first PCR, forward primer
FLAG-AVPR2_fwd	GACTACAAGGACGATGATGAC-GCCAGCATCGA-TATGCTCATGGCGTCCACC	AVPR2	Addition of signal peptide, first PCR, forward primer
FLAG-GCGR_fwd	GACTACAAGGACGATGATGAC-GCCAGCATCGA-TATGCCCCCCTGCCAG	GCGR	Addition of signal peptide, first PCR, forward primer
FLAG-GLP1R_fwd	GACTACAAGGACGATGATGAC-GCCAGCATCGA-TATGGCCGGCGCCCC	GLP1R	Addition of signal peptide, first PCR, forward primer
PCR_attB2_rev	GGGGACCACTTTGTACAA-GAAAGCTGGGTC	AVPR2, DRD1, DRD2, HTR2A, GCGR	Addition of signal peptide, first & second PCR, reverse primer
GLP1R_attB2_rev	GGGGACCACTTTGTACAA-GAAAGCTGGGTCGCTGCAG-GAGGCCTGG	GLP1R	Addition of signal peptide, first & second PCR, reverse primer
attB1-HA1-SP-FLAG_fwd	GGGGACAAGTTT-GTACAAAAAAGCAGGCTCCAC-CATGAAGAC-GATCATCGCCCTGAGC-TACATCTTCTGCCTGGTATTCGC CGACTACAAGGAC-GATGATGACGCC	All	Addition of signal peptide, second PCR, forward primer
